# The Effect of Physical Exercise Training on Neck and Shoulder Muscle Function Among Military Helicopter Pilots and Crew: A Secondary Analysis of a Randomized Controlled Trial

**DOI:** 10.3389/fpubh.2020.546286

**Published:** 2020-11-23

**Authors:** Mike Murray, Britt Lange, Karen Søgaard, Gisela Sjøgaard

**Affiliations:** ^1^Department of Sports Science and Clinical Biomechanics, University of Southern Denmark, Odense, Denmark; ^2^Department of Anesthesia and Intensive Care Medicine, Odense University Hospital, Odense, Denmark; ^3^Department of Clinical Research, University of Southern Denmark, Odense, Denmark

**Keywords:** neck, exercise, intervention, muscle strength, rate of force development, musculoskeletal pain

## Abstract

**Introduction:** This study presents secondary outcome analyses, in terms of muscle function [i.e., maximal voluntary contraction (MVC) and rate of torque development (RTD)] from a parallel group, single blinded, randomized controlled trial introducing a physical exercise training intervention aiming to reduce neck pain among military helicopter pilots and crew-members.

**Methods:** Participants (50 pilots, 58 crew-members) were recruited from the Royal Danish Air Force and randomized to either an exercise-training-group (ETG; *n* = 35) or a reference-group (REF; *n* = 34). Participants in ETG received 20 weeks of self-administered exercise training specifically tailored to target the neck and shoulder muscles. REF received no training. Outcome: (1) MVC was measured for cervical extension and flexion as well as shoulder elevation and abduction, (2) RTD was measured for cervical extension and flexion. Adherence to training was self-reported and categorized as regular if performed at least once a week.

**Results:** MVC for cervical extension was significantly increased at follow-up in ETG (37.5 ± 11.2 Nm at baseline, change: 2.1 ± 8.3 Nm) compared to REF (38.1 ± 10.7 Nm at baseline, change: −2.4 ± 6.8 Nm) according to intension-to-treat analysis (*p* = 0.018*)*. Likewise, RTD was significantly increased in ETG for cervical extension (149.6 ± 63.3 Nm/s at baseline, change: 14.7 ± 49.0 Nm/s) compared to REF (165.4 ± 84.7 Nm/s at baseline, change: −16.9±70.9 Nm/s) (*p* = 0.034*)*. The cervical extension/flexion MVC-ratio was significantly different at follow-up (*p* = 0.039) between ETG (1.5 ± 0.5 at baseline, change: −0.0 ± 0.3) compared to REF (1.5 ± 0.5 at baseline, change: −0.2 ± 0.4). Per-protocol analysis of MVC, including only participants in ETG with regular training adherence (*n* = 10), showed a significant increase for cervical extension (33.2 ± 7.3 Nm at baseline, change: 6.0 ± 5.4 Nm) and shoulder elevation right side (143.0 ± 25.8 Nm at baseline, change: 15.8 ± 18.1 Nm).

**Conclusion:** Physical exercise training significantly improved MVC and RTD in the upper neck extensors. Only approximately 1/3 of participants in ETG adhered to training regularly, which likely attenuated the effectiveness of the training intervention on neck and shoulder muscle function. Future studies should focus on the practical implementation of self-administered exercise training to improve adherence.

## Introduction

Neck pain is documented as highly prevalent within military helicopter communities (1–4). A Canadian survey reported that up to 81% of the surveyed helicopter pilots and 85% of the crew-members had experienced neck pain related to helicopter flights (5). Neck pain within the helicopter community is an important issue to address, but limited research has been conducted aiming to prevent the high prevalence of neck pain within this occupational group. Different aspects of helicopter flight and factors associated with neck pain among helicopter pilots and crew-members have been assessed (1). One factor often associated with neck pain and discomfort is the use of night vision goggles (NVG) (5). Studies conducted in laboratory settings have established that the helmet mass increase the metabolic response (6, 7) and muscle strain (8) in the cervical musculature. Muscle strain is also affected by adapted postures during flight, and studies have found positioning of the head and body to have greater influence on muscle strain than the load due to head-worn equipment such as NVG (9, 10). Recently, we addressed this issue during real flight scenarios (11). External loading on the cervical spine, by use of a helmet and NVG, may potentially evolve into excessive internal loading of the cervical vertebrae and the musculature supporting the neck. This might translate into the high prevalence of neck pain observed within the helicopter community.

Studies on patients with chronic neck pain have reported significant reductions in maximal isometric strength for cervical flexion (12) and cervical extension (13), or in both (14), as compared to healthy matched controls, with the greatest reduction seen in the extensor muscle groups (15). Selective impaired neck muscles strength in either flexor of extensor muscles may impact the normal balance between cervical extension and flexion strength, which among pain free individuals has been found to be approximately 1.7 (16). The extensive load on the upper neck extensors during flight may in particular call for proper cervical extension strength and an extension/flexion ratio of 1.7 or more (11). Rapid movements have been found to exacerbate fear of pain among patients with chronic pain (17, 18). In addition, rapid force development of painful muscles and pain-free synergistic muscles was also found to be more severely impaired among individuals with chronic musculoskeletal pain than maximal strength capacity (19). Pain may therefore not only impact isometric maximal force development but also the speed by which the movement can be performed (20). Physical exercise training may be beneficial in terms of pain development prevention and rehabilitation by means of increasing individual capacity and thereby lowering the relative workload (21).

Reduction in work related neck pain among a number of different working populations has been found using all-round physical exercise training (22), proprioceptive muscle coordination training (23), and in particular strength training (24–27). This was confirmed for office workers in a recent systematic review and meta-analysis (28). However, another systematic review of such training interventions reports uncertainty regarding the effectiveness of exercise in the relief of neck pain (29). Therefore, knowledge regarding effectiveness of exercise on neck pain within specific occupational groups still needs to be addressed. For instance, such evidence is needed in order to establish specific guidelines on physical exercise training for the prevention or rehabilitation of flight related neck pain within the helicopter community. This paper presents secondary outcome analyses, in terms of muscle strength, from a randomized controlled trial introducing a physical exercise training intervention aiming to reduce and prevent neck pain among military helicopter pilots and crew-members (30). At baseline the 12-month prevalence of neck pain was 82 and 90% for crew and pilots, respectively, and around 1/3 had experienced pain 8–30 days. Pain may lead to flying restrictions and jeopardize future employment opportunities thus legitimizing interventions such as strength training that may reduce such pain. Of interest was further if such training could also improve relevant physical capacities. The hypotheses were that the adherence to a self-administered physical exercise training intervention would: (1) significantly increase neck and shoulder maximal voluntary contraction (MVC), and rate of torque development (RTD), and (2) significantly increase MVC and RTD during cervical extension and flexion, maintaining a balanced extension/flexion MVC-ratio.

## Methods

### Study Design

This study was a parallel group, single blinded, randomized-controlled trial, including baseline and follow-up measurements after 20 weeks. The study was conducted within the Royal Danish Air Force (RDAF) from November 2013 to April 2014 and the study was approved by the local Ethics Committee of Southern Denmark (S-20120121) and qualified for registration in ClinicalTrails.gov (NCT01926262). Each subject provided written informed consent before participation.

### Participants and Randomization

**Figure 1:**
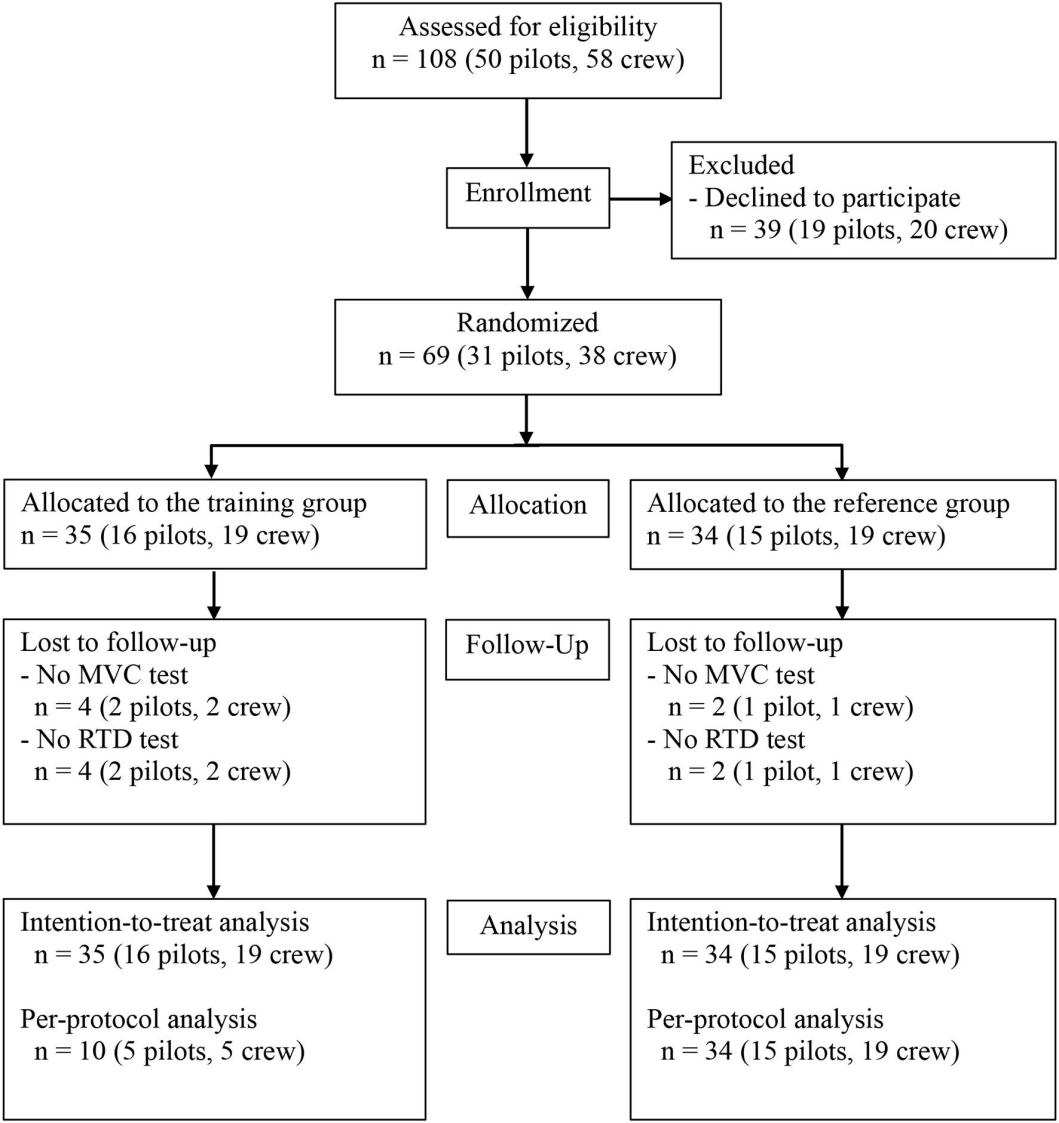
Flow of participants.

In total, 50 military helicopter pilots and 58 crew-members, from two squadrons within the RDAF were invited to participate in this study. After oral and written information regarding the study, informed consent was obtained from 69 participants (31 pilots—hereof 2 females, 38 crew-members—all males). Participant flow is depicted in Figure 1. Inclusion criteria were: (1) occupation as a helicopter pilot or crew-member (technician, systems operator, tactical helicopter observer, and/or navigator), (2) operational flight status at enrollment, (3) operational flying within the previous 6 months. Exclusion criteria were: (1) participation in a training intervention within the last 12 months. Participants were assigned a random identification number at enrollment and randomized 1:1 to either an exercise-training-group (ETG) or a reference-group (REF). The randomization procedure was performed after baseline assessments. A detailed description can be found elsewhere (30).

### Exercise Intervention

Participants randomized to REF received no training, but were encouraged to continue with their usual exercise activities. Participants in the ETG received 20 weeks of strength, endurance, and coordination training, specifically tailored to target the neck and shoulder muscles based on work exposure assessments (11). Training was based on self-management education and was to be performed three times 20 min a week within working hours. Every training session was initiated with one or two conditioning exercises for the neck, specifically targeting the deep cervical musculature. Exercises included: Upper cervical flexion/extension from a supine position, and cervical rotation against mild resistance. The conditioning exercise was followed by training exercises for the neck targeting larger muscle groups. Exercises included: cervical extension, cervical flexion (straight forward and in oblique directions), and lateral flexion. Lastly, participants performed two training exercises for the shoulders including shrugs and reverse flyes. Training exercises for the neck and shoulders were performed using elastic training bands for resistance (Thera-Band®, The Hygenic Corporation, USA) and a head harness (The Original Neck Flex® Head Harness, Gonzo Companies, USA). The training program was designed with systematic variation in intensity and volume based on undulating (non-linear) periodization securing a progressive overload. Sessions ranged between 2 and 4 sets and training intensity ranged between 12 and 20 repetitions over the 20 weeks of training. This has previously for each of the 20 weeks of training been described in details in the protocol for the study (30).

The training program was evidence based (31, 32) designed by an interdisciplinary team of sports exercise training specialists, physiotherapists, doctors and chiropractors. A complete exercise description has been published elsewhere (30).

### Outcome Measurements

Participant characteristics, including age, height, seated height, weight and neck circumference were measured with standard clinical rulers and measuring tapes both pre and post intervention. Measurements of muscle function included MVC and RTD for bilateral shoulder abduction and elevation, as well as cervical extension and flexion. Measurements were performed following 10 min of warming up on a rowing ergometer. All measurements have been described in detail previously (30) and will only be described briefly. During MVC for shoulder abduction participants were positioned seated with both arms held close to the body and elbows flexed 90 degrees. Two force transducers (load cell, KIS-2, 2kN, Vishay Nobel, Vishay Precision Group, USA) were positioned 1 cm above the lateral epicondyle. The lever arm between the lateral edge of acromion and the force transducers was used for later analysis. During MVC measurements for shoulder elevation, a force transducer was placed on each shoulder 1 cm medially from the lateral edge of the acromion. The lever arm was measured from the seventh cervical vertebra to the center of the transducers. During MVC and RTD for cervical extension and flexion the participants were positioned seated with their backs straight, arms positioned along the sides of the body, both feet on the floor, and head and neck held in an anatomical neutral position. Participants were positioned with their backs against the experimental set-up during cervical extension and the force transducers were positioned just above the external occipital protuberance. During cervical flexion participants were positioned with their front against the experimental set-up and a force transducer was positioned just above the eyebrows. The vertical distance between the seventh cervical vertebra and the center of the force transducer was measured as the lever arm. All MVC values were calculated as torque and presented in Nm. Regarding the cervical extension/flexion MVC-ratio the data were also calculated based on the values in N.

Before testing, subjects were strapped firmly into place using belts and MVC and RTD were measured using a standardized method and procedure (33). The instruction for participants during the MVC tests was to increase the force gradually during measurements reaching MVC in 5 s, hold the force at MVC for 2 s and slowly reduce the force again. A minimum of three MVC tests were performed. If the result of the third MVC was ≥5% compared to the first or second MVC, another MVC trial was performed. A maximum of five trials were allowed for each test. The MVC tests were performed with verbal encouragement. Force was amplified with a gain of 100 (National Instruments Corporation, Full bridge amplifier, SCC-SG24, USA), and sampled at 100 Hz using a 16-bit A/D converter (National Instruments Corporation, DAQ Card TM-6034E, USA). The MVC was determined as the peak torque (unit Nm) and the highest MVC value of all trials was saved and stored for analysis. For cervical extension and flexion, the MVC-ratio was calculated as MVC for cervical extension divided with MVC for cervical flexion. RTD was measured during MVC for shoulder elevation, cervical extension and flexion. For these trials the instruction to participants was: “on the command 3-2-1 you must apply a slight pressure against the force transducer and on the command NOW… press as hard and fast as possible. You must keep the pressure for a second and then slowly relax again” (30). Force was amplified with a gain of 100 and sampled at 1000 Hz using the A/D converter. A total of three RTD trials were performed. For each trial the RTD (unit Nm/s) was determined as the steepest slope over 100 ms of the rising part of the filtered torque-time curve. The highest obtained value was determined as the peak RTD.

## Statistical Analysis

Normality of the residuals was assessed using a *Q*–*Q* plot, and a Shapiro Wilk's test and showed no consistent deviation from a normal distribution. Participant characteristics: age, height, seated height, weight, neck circumference and lever arms for shoulder abduction, shoulder elevation, cervical flexion, and cervical extension, were analyzed for between-group-difference at baseline using the Student's *t*-test. Between-group-differences for MVC and RTD were analyzed using delta values (change from pre- to post-intervention) using the Student's *t*-test. Within-group-changes were analyzed using a paired *t*-test. Two analyses were conducted: (1) an intention-to-treat analysis (ITT-analysis) including all randomized participants, and (2) a per-protocol analysis (PP-analysis) only including participants in ETG with regular training adherence defined as at least 1 training session a week throughout the 20-week intervention period (30). Missing data was imputed using last observation carried forward or backwards. When missing at both baseline and follow-up, baseline values were imputed as the mean value of the entire cohort and values at follow-up were imputed at the baseline value adjusted for the observed change (%) among those measured in ETG or REF, respectively. Results are presented as mean ± SD if not otherwise specified. The level of statistical significance was *p* < 0.05. Statistical analyses were performed in Stata Statistics/Data Analysis version 14.0 (StataCorp LP, USA).

## Results

### Pre-intervention

No significant between-group-differences were found at baseline regarding participant characteristics (Table 1). Measurements for MVC and RTD were also not significantly different between groups at baseline (Table 2). The pre-intervention extension/flexion MVC-ratio was: 1.5 ± 0.5 Nm in ETG and 1.5 ± 0.5 Nm in REF with no significant difference between groups (*p* = 0.494). The MVC-ratio based on calculations without lever arm measurements, was: 1.4 ± 0.5 N in ETG and 1.5 ± 0.5 N in REF with no significant difference between groups (*p* = 0.632).

**Table 1:** Participant's baseline characteristics and lever arm length used for torque measurements.

	**ETG (*n* = 35)**	**REF (*n* = 34)**
Age (years)	40.4 ± 6.7	40.7 ± 8.4
Height (m)	1.82 ± 0.07	1.80 ± 0.08
Seated height (cm)	94.5 ± 4.5	94.5 ± 4.1
Weight (kg)	84.2 ± 12.7	83.7 ± 11.8
Neck circumference (mm)	390 ± 24	391 ± 20
Lever arm: cervical extension (mm)	151 ± 17	158 ± 13
Lever arm: cervical flexion (mm)	148 ± 17	154 ± 16
Lever arm: shoulder elevation (right) (mm)	182 ± 18	174 ± 12
Lever arm: shoulder elevation (left) (mm)	184 ± 17	175 ± 14
Lever arm: shoulder abduction (right) (mm)	274 ± 17	267 ± 31
Lever arm: shoulder abduction (left) (mm)	280 ± 26	269 ± 19

*Values are presented as mean and standard deviation*.

**Table 2:** Intention-to-treat analysis of maximal voluntary contraction and rate of torque development.

			**ETG (*n* = 35)**	**REF (*n* = 34)**	***P*-value**
Rate of torque development	Cervical extension (Nm/s)	Baseline	149.6 ± 63.3	165.4 ± 84.7	0.384
		Follow-up	164.3 ± 73.4	148.4 ± 64.9	0.343
		Change	14.7 ± 49.0	−16.9 ± 70.9	0.034^*^
	Cervical flexion (Nm/s)	Baseline	104.0 ± 47.7	109.1 ± 49.5	0.665
		Follow-up	115.2 ± 57.0	104.0 ± 40.9	0.351
		Change	11.2 ± 46.7	−5.1 ± 47.3	0.153
Maximal voluntary contraction	Cervical extension (Nm)	Baseline	37.3 ± 11.2	38.1 ± 10.7	0.747
		Follow-up	39.3 ± 10.2	35.8 ± 10.3	0.153
		Change	2.1 ± 8.3	−2.4 ± 6.8^†^	0.018^*^
	Cervical flexion (Nm)	Baseline	27.5 ± 9.8	26.5 ± 8.4	0.671
		Follow-up	28.6 ± 9.9	27.0 ± 7.0	0.428
		Change	1.2 ± 6.4	0.5 ± 4.3	0.595
	Shoulder elevation (right) (Nm)	Baseline	143.5 ± 39.2	135.9 ± 30.8	0.374
		Follow-up	149.1 ± 40.4	134.8 ± 32.2	0.108
		Change	5.6 ± 21.5	−1.1 ± 20.5	0.188
	Shoulder elevation (left) (Nm)	Baseline	154.3 ± 45.8	142.6 ± 33.7	0.231
		Follow-up	150.7 ± 45.3	137.2 ± 35.1	0.175
		Change	−3.6 ± 16.3	−5.3 ± 15.7	0.662
	Shoulder abduction (right) (Nm)	Baseline	103.2 ± 28.4	108.1 ± 30.7	0.485
		Follow-up	104.9 ± 33.7	109.5 ± 24.3	0.528
		Change	1.8 ± 20.5	1.3 ± 18.2	0.917
	Shoulder abduction (left) (Nm)	Baseline	106.8 ± 31.7	108.6 ± 33.4	0.827
		Follow-up	104.3 ± 36.6	109.9 ± 27.2	0.480
		Change	−2.5 ± 20.8	1.3 ± 14.8	0.387

Values are presented as mean and standard deviation. Significant between-group-differences (^*^). Significant within-group-differences (^†^).

### Post-intervention (ITT-Analysis)

#### Training Adherence

In the ETG 25 out of 35 participants (71%) returned the post-intervention questionnaire regarding training adherence as previously reported (34). Among all participants in the ETG, 10 participants (29%) (5 pilots and 5 crew-members) reported having trained regularly 1–3 times a week throughout the intervention period, 9 participants (26%) reported having trained irregularly, but at least 2–4 times a month, 5 participants (14%) reported that they had done some training but stopped training after a while, and 1 participant (3%) did not use the training offer.

#### MVC and RTD

At follow-up, a significant between-group-difference was found for change in MVC during cervical extension (Table 2). Furthermore, RTD during cervical extension also increased significantly in ETG as compared to REF (Table 2). No significant difference was observed for change in cervical flexion, shoulder abduction (right/left) or shoulder elevation (right/left) at post-intervention, according to the ITT-analysis. Within the REF group a significant reduction for MVC during cervical extension was found (Table 2). Results for MVC are presented in Nm but were also analyzed in N and showed the same significant between-group-differences. Also, no significantly different results were found when RTD was analyzed using N/s compared to Nm/s. Measurements of the lever arms used are depicted in Table 1. No significant difference in neck circumference was found post-intervention between ETG and REF (ETG, change: −1.0 ± 11 mm vs. REF, change: −6.0 ± 11 mm) (*p* = 0.119). A significant reduction in neck circumference within REF was present (391 ± 20 mm at baseline, change: −6.0 ± 11 mm) (*p* = 0.006). No significant within-group-change for neck circumference was observed for ETG.

#### Cervical Extension/Flexion MVC-Ratio

A significant difference in change of MVC-ratio was present between groups with the intervention (ETG, change: 0.0 ± 0.3 vs. REF, change −0.2 ± 0.4) (*p* = 0.039). The difference was also significant when the MVC-ratio was calculated without lever arm measurements (ETG, change: 0.0 ± 0.3 vs. REF, change: −0.2 ± 0.4) (*p* = 0.049). Within REF, the reduction in MVC-ratio was significant from pre- to post-intervention based on Nm calculations (from: 1.5 ± 0.5 to: 1.4 ± 0.4) (*p* = 0.007), and also based on N calculations (from: 1.5 ± 0.5 to: 1.3 ± 0.3) (*p* = 0.012).

### Post-intervention (PP-Analysis)

**Figure 2:**
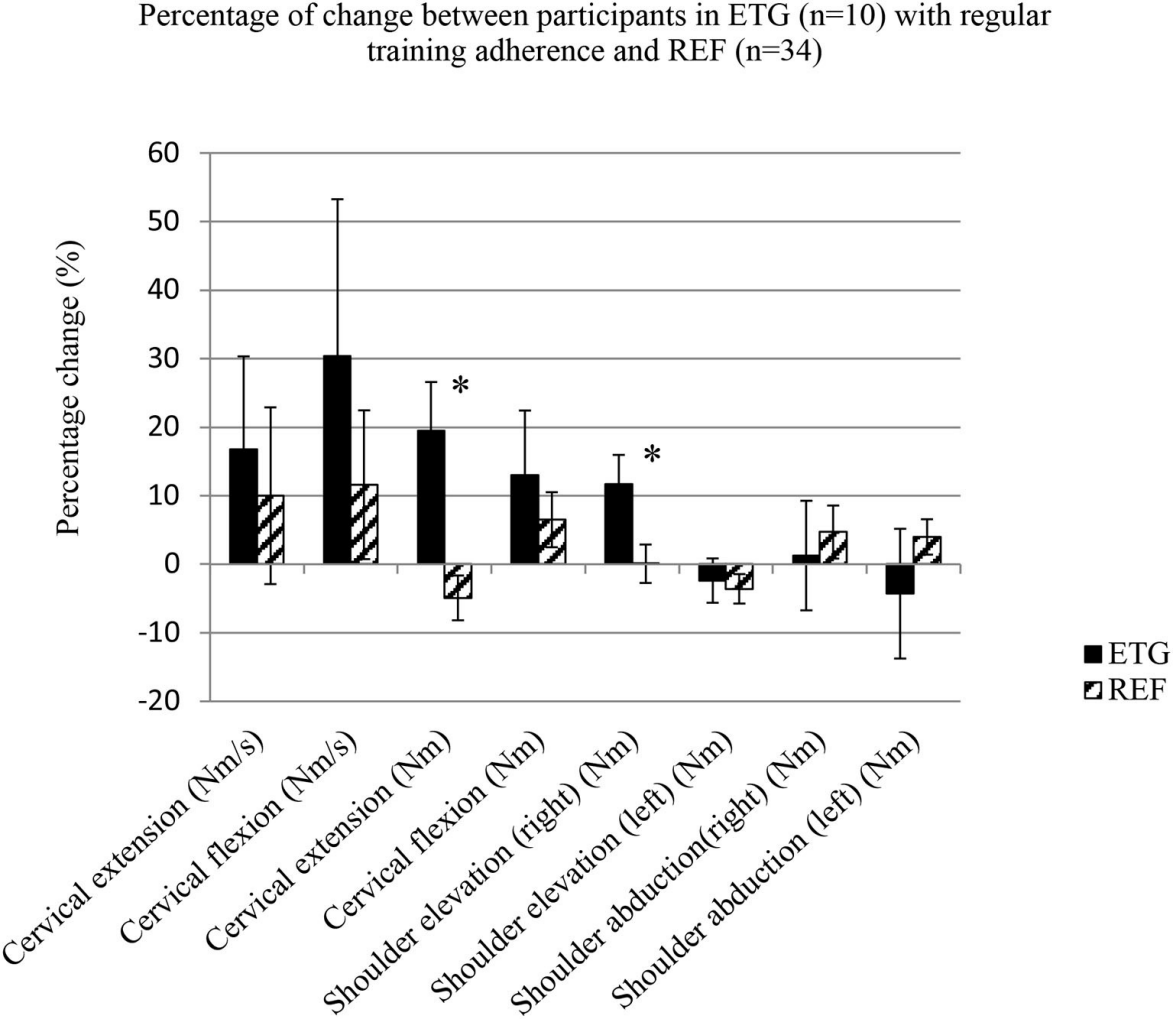
Per-protocol analysis as percentage of change for maximal voluntary contraction and rate of torque development. Values are presented in mean and standard error. Exercise-training-group (ETG; *n* = 10). Reference-group (REF; *n* = 34). Significant between-group-difference (*).

Per-protocol-analysis included only participants from the ETG with regular training adherence (*n* = 10) vs. all participants in the REF group. Significant between-group-differences were present regarding change of MVC for cervical extension (change: 6.0 ± 5.4 Nm, vs. −2.4 ± 6.8 Nm), and shoulder elevation (right side) (change: 15.8 ± 18.1 Nm vs. −1.1 ± 20.5 Nm) (Table 3). Between-group-changes are presented in Figure 2 as percentage of change. Within-group-changes for MVC in ETG were significant for cervical extension (33.2 ± 7.3 Nm at baseline, change: 6.0 ± 5.4 Nm) (*p* = 0.007*)*, and for shoulder elevation (right side) (143.0 ± 25.8 Nm at baseline, change: 15.8 ± 18.1 Nm) (*p* = 0.022*)*. No significant difference for change in neck circumference was observed between ETG and REF. No significant difference was observed for the cervical extension/flexion MVC-ratio between ETG (change: 0.0 ± 0.4) and REF (change: −0.2 ± 0.4) (*p* = 0.122). The non-significant difference persisted when the MVC-ratio was analyzed without lever arm measurements (ETG, change: 0.0 ± 0.4 vs. REF, change: −0.2 ± 0.4) (*p* = 0.128).

**Table 3:** Per-protocol analysis of maximal voluntary contraction and rate of torque development.

			**ETG (*n* = 10)**	**REF (*n* = 34)**	***P*-value**
Rate of torque development	Cervical extension (Nm/s)	Baseline	139.6 ± 50.0	165.4 ± 84.7	0.366
		Follow-up	162.4 ± 71.5	148.4 ± 64.9	0.562
		Change	22.8 ± 51.1	−16.9 ± 70.9	0.107
	Cervical flexion (Nm/s)	Baseline	99.8 ± 36.9	109.1 ± 49.5	0.588
		Follow-up	116.3 ± 60.3	104.0 ± 40.9	0.460
		Change	16.4 ± 72.0	−5.1 ± 47.3	0.270
Maximal voluntary contraction	Cervical extension (Nm)	Baseline	33.2 ± 7.3	38.1 ± 10.7	0.181
		Follow-up	39.2 ± 8.5	35.8 ± 10.3	0.345
		Change	6.0 ± 5.4^†^	−2.4 ± 6.8^†^	0.001^*^
	Cervical flexion (Nm)	Baseline	25.1 ± 9.7	26.5 ± 8.4	0.657
		Follow-up	26.7 ± 7.4	27.0 ± 7.0	0.897
		Change	1.5 ± 5.9	0.5 ± 4.3	0.527
	Shoulder elevation (right) (Nm)	Baseline	143.0 ± 25.8	135.9 ± 30.8	0.512
		Follow-up	158.7 ± 29.7	134.8 ± 32.2	0.042
		Change	15.8 ± 18.1^†^	−1.1 ± 20.5	0.024^*^
	Shoulder elevation (left) (Nm)	Baseline	157.0 ± 33.7	142.6 ± 33.7	0.240
		Follow-up	151.8 ± 28.1	137.2 ± 35.1	0.238
		Change	−5.2 ± 16.3	−5.3 ± 15.7	0.987
	Shoulder abduction (right) (Nm)	Baseline	104.2 ± 31.7	108.1 ± 30.7	0.725
		Follow-up	102.9 ± 33.9	109.5 ± 24.3	0.495
		Change	−1.3 ± 32.3	1.3 ± 18.2	0.738
	Shoulder abduction (left) (Nm)	Baseline	103.2 ± 29.0	108.6 ± 33.4	0.648
		Follow-up	95.9 ± 33.1	109.9 ± 27.2	0.181
		Change	−7.3 ± 34.2	1.3 ± 14.8	0.252

Values are presented as mean and standard deviation. Significant between-group-difference (^*^). Significant within-group-difference (^†^).

## Discussion

The main findings of this study were that: (1) 20 weeks of physical exercise training, designed to reduce and prevent neck pain, significantly improved MVC and RTD in the cervical extensor muscles of military helicopter pilots and crew-members, (2) participants with regular training adherence additionally increased their MVC for shoulder elevation in the right side significantly, and (3) the physical exercise intervention proved preventive in terms of maintaining the cervical extension/flexion MVC-ratio, that decreased significantly in REF from baseline to follow-up.

### MVC and RTD

In agreement with our first hypothesis, self-administered physical exercise training resulted in significant changes between groups at follow-up. The overall difference between groups regarding MVC for cervical extension was ~11% with an increase of ~5% in ETG and a decrease of ~6% in REF. The overall difference in RTD during cervical extension amounted to ~20% with an increase of ~10% in ETG and a decrease of ~10% in REF. Helicopter pilots and crew-members may potentially benefit from increasing upper neck muscle strength, as improvements in strength would increase individual capacity and potentially reduce the relative workload on cervical musculature during flight (21). The ability to develop a fast force torque response to resist external loading may be important, as this will provide neck stabilization and prevent overload of neck tissue. Increasing MVC and RTD may therefore be of functional importance. The decrease in MVC and RTD observed in REF may be due to seasonal variation in work exposure. The winter period incorporates many flight hours with NVG as daylight is short, and pilots and crew-members may experience deterioration in muscle function during the winter period influenced by an excessive workload due to NVG use (11). This could also explain the significant reduction in neck circumference observed in REF, but not in ETG. If the reduction in REF is due to seasonal variation in work exposure, it would be especially important for pilots to engage in regular exercise training in preparation for the winter period. Physiological adaptions in response to exercise training are related to the specific characteristics of the exercises and stimuli used (35). This phenomenon is also referred to as the principle of specificity, underlining that the greatest improvements in muscle function will be found using a test protocol that reflects training mode (35). Our exercise training program included a high amount of repetitions maximum (12–20 RM) with between 2 and 4 sets. Only brief pauses between sets were incorporated to stimulate an increase in endurance to a greater extent than increased strength (30). This decision was based on a previous in-flight exposure assessment where electromyography recordings were used (11) and demonstrated prolonged activation of the neck/shoulder muscles. These former findings imply that neck/shoulder muscles might also benefit from endurance training and not strength training alone (1). Thus, our training program was designed to improve strength—endurance, and this should be taken into consideration when evaluating the improvements in MVC and RTD.

### Adherence

It is important to take into account training adherence in the interpretation of our results. Participants adhering to training regularly gained the largest increase in MVC regarding cervical extension of ~18% in ETG, as compared to a reduction of ~6% in REF. A significant increase in MVC in the right shoulder of ~11% was also found among participants who adhered to regular training, as compared to a reduction of ~1% in REF. The small magnitude of effect in our results may be caused by the low training adherence, as only 29% within ETG trained with a frequency of ≥1 day/week throughout the intervention period. This is low compared to previous exercise interventions with adherence rates of 53–77% found in studies on helicopter pilots and crew-members (36, 37). Furthermore, it must be underlined that only 25 out of 35 participants in ETG responded on the questionnaire regarding training adherence. Our adherence analyses are therefore based on roughly 2/3 of the ETG group. Self-reported adherence to training has been found reliable compared to actual registration of training participation (24). We used a cut-point of performing at least 1 training sessions a week as being regular and sufficient stimulus for physiological adaptions to occur (35). The same cut-point has previously been used (27). The low level of adherence in the present study is expected to have impacted on the effectiveness of the exercise intervention. Still, observed MVC difference according to the PP-analysis of ~24% for cervical extension and ~12% for shoulder elevation in the right side indicates that our exercise intervention was effective when performed regularly.

### The General Strength of Aircrew

Pilots and crew-members are exposed to some of the highest physical demands within the RDAF and undergo annual health and fitness evaluations (27). Pilots and crew-members are physically fit and healthy individuals, and accordingly, a potential strength gain from an exercise intervention would be expected to be lower compared to that of untrained individuals. This is supported when comparing our results with findings by Faber et al. (38), who reported MVC values for shoulder abduction of 67 Nm (dominant side) and 71 Nm (non-dominant side), and 130 Nm and 126 Nm for shoulder elevation, respectively, among gender- and age-matched subjects in Denmark with different work occupations. In the present study, values for shoulder abduction were ~35% higher, and for shoulder elevation ~10% higher (depending on dominant side). These results show that pilots and crew-members are stronger in the shoulder musculature as compared to the general working population. In contrast, with regards to neck muscle strength, Jordan et al. (39) reported MVC values for cervical extension of around 55 Nm and cervical flexion of around 30 Nm for a large gender-matched Danish non-pilot population, with the present values being ~10 and ~30% lower. This is somewhat surprising, but the lack of superior neck muscle strength in our study group is supported by results by Seng et al. (40) who reported no significant difference between fighter aircraft pilots and non-pilots in neck muscle strength, calculated as MVC in neck extension, flexion, as well as left and right lateral bending. However, these results are not supported by Alricsson et al. (41), who reported a significantly higher level of muscle strength among Swedish air force jet pilots equal to ~9% during cervical extension (65 Nm) and ~31% during cervical flexion (47 Nm), as compared to a reference group of young conscripts doing their military service (59 Nm and 36 Nm). Thus, overall discrepancies regarding the cervical strength of pilot compared to the non-pilot populations are present. Likewise, discrepancies were found between previously published results from helicopter pilots and crew-members compared with our results with regards to cervical muscle strength. Ang et al. (42) previously reported values of MVC for cervical extension to be ~38% (52 Nm) higher, and flexion to be ~6% (29 Nm) higher as compared to corresponding values in our results. Furthermore, Van den Oord et al. previously published results of cervical extension and cervical flexion including both asymptomatic and symptomatic pilots and rear-aircrew with no significant difference between groups. Compared to our study, results from Van den Oord et al. (43) were ~45% (55 Nm) higher for pilots and ~60% (60 Nm) higher for crew-members during cervical extension, whereas cervical flexion for pilots was found to be ~17% (23 Nm) lower, and ~26% (22 Nm) lower for crew-members in comparison to our findings. Overall, our values are lower than those previously reported. However, that does not impact on the main finding of this study regarding changes in strength with training, since the same test procedure was used at baseline and follow-up.

Comparing results of cervical strength between studies may be challenging due to the use of different methods and protocols for quantifying cervical strength (44). In the study by Jordan et al. (39), participants trained on the measuring apparatus prior to the final tests in a protocol with light resistance (women: 2–3 kg in flexion and 3–4 kg in extension, men: 4–5 kg in flexion and 6–7 kg in extension), with 6–7 repetitions in each direction, to familiarize participants with the procedure, potentiate involved muscles, and overcome fear avoidance. The use of a familiarization procedure may have led to higher values in the study by Jordan et al. Lastly, participants were not strapped during the test procedure, but were instructed to grip onto armrests to maintain their position during measurements (39). The larger degree of freedom and arm placement may also have proven beneficial in terms of higher force values, as compared to our test protocol. We recognize that the reliability of MVC results between studies might be subject to methodological differences. However, based on an overall assessment of our results in addition to the above mentioned studies, aircrew may be considered stronger in the shoulder musculature, but equally strong during cervical extension (38–60 Nm) and flexion (22–27 Nm), as compared to a non-pilot population (39). This is an important finding, since pilots and crew-members must wear helmets and additional helmet mounted equipment that place considerable strain on their cervical musculature during flight (11). Enhancing upper neck muscle function may reduce the relative load with potential impact on the high prevalence of neck pain observed within this occupational group.

### Cervical Extension/Flexion MVC-Ratio

In agreement with our second hypothesis, the physical exercise intervention maintained the cervical extension/flexion MVC-ratio in the ETG group while the MVC-ratio was significantly decreased in REF from pre- to post-intervention as a result of a significant decline in MVC in cervical extension. Suryanarayana et al. (16) and Jordan et al. (39) both found a MVC-ratio of 1.7 to be the average among healthy individuals. In comparison, our MVC-ratio was slightly lower, and this may underline that pilots need to specifically address neck muscle training in order to maintain a normal strength relationship between cervical extension and flexion. The posterior neck muscles have a larger physiologic cross-sectional area compared to the anterior neck muscles (45) and should therefore be capable of higher force development. The significant decrease in MVC-ratio in the REF group compared to the ETG group may be important in relation to the risk of neck pain development. Cervical pain has been reported to influence MVC measurements in a number of individual studies of non-pilot populations (12, 14, 46–49). However, conflicting results have been reported in this regard, as Ang et al. (42), who compared MVC-measures between helicopter pilots with frequent neck pain episodes and helicopter pilots without pain, found no significant MVC differences. These findings are supported by Van den Oord et al. (43), who published MVC results on cervical extension and cervical flexion including both asymptomatic and symptomatic pilots and rear-aircrew, and reported no significant MVC differences. Based on the above mentioned relations, it may be questionable whether pain inhibition during MVC testing is directly comparable between military- and patient-populations. From a functional point of view, it would seem beneficial especially for helicopter pilots and crew-members to improve muscular capacity in the cervical extensors, as this muscle region has been found highly active during flight (11). The MVC-ratio may be used as a guideline for future training modalities, in order to individualize and balance training programs further in this occupational group.

### Limitations and Strengths

The limitation of this study was the low adherence to self-administrated exercise training. Further research is requested to identify ways to improve such training because supervised training is not possible in all job categories. The strengths of the study were the rigid randomized controlled design and the intervention protocol consisting of validated training exercises. Likewise, the possibility of performing a per protocol analysis based on recordings of regular adherence is a strength, because this supported the training exercises to be effective if performed.

### Implications of Study Findings to Research and Practice

Specific exercise training targeting the neck and shoulder muscles can improve muscle strength and function that may combat muscle disorders among workers exposed to high physical loadings in the neck/shoulder region. Regular adherence to training is decisive for positive effects. If self-administrated training is the optimal choice due to, e.g., job specific logistics it is particularly important to identify means for attaining a high adherence.

## Conclusion

Specific exercise training targeting the neck and shoulder muscles significantly improved MVC and RTD in the upper neck extensors of participants in the ETG. Approximately 1/3 of participants in ETG adhered to regular training, and this is likely to attenuate the effectiveness of the training intervention on neck and shoulder muscle function. This is underlined by an additional increase in MVC for the right shoulder among participants with regular training adherence. The MVC results for pilots and crew-members were above population mean values for shoulder strength, but equal to such values for neck muscle strength. To accommodate job specific loading of cervical musculature during flight, pilots and crew-members should engage in regular exercise training of the neck muscles. Further, future studies should focus on the practical implementation of self-administered exercise training to improve adherence.

## Data Availability Statement

The raw data supporting the conclusions of this article will be made available by the authors, without undue reservation.

## Ethics Statement

The studies involving human participants were reviewed and approved by The Regional Committees on Health Research Ethics for Southern Denmark (S-20120121). The patients/participants provided their written informed consent to participate in this study.

## Disclosure

This study was financially supported by the Royal Danish Air Force and the University of Southern Denmark. There are no conflicting financial interests between institutions or between the authors of this paper.

## Author Contributions

MM together with GS, KS, and BL were responsible for the design of the study. MM performed the measurements, analyzed the data, and drafted the manuscript. All authors have made significant intellectual contributions to the manuscript and approved the final version before publication.

## Conflict of Interest

The authors declare that the research was conducted in the absence of any commercial or financial relationships that could be construed as a potential conflict of interest.
